# Labial Support

**Published:** 1898-08

**Authors:** J. D. Bradley

**Affiliations:** Kingston, Ga.


### LABIAL SUPPORT.

BY J. D. BRADLEY, M. D., KINGSTON, GA.

Presented to the Alumni Association of the Sounthern Medical College.

To prevent laceration of the perineum has long been a complex question with me, hence I have adopted the following measures in common deliveries.

I place myself on the right with the patient in the dorsal position, and when the head is well descended so as to distend the perineum, with my left hand I press forcibly on the labia majora, with my thumb and index finger approximating the parietal protuberances of the fœtus. By making this pressure downwards and forwards it carries the soft structures from the mons veneris and round about the anterior commissure. This gives an increase to the antero-posterior diameter of the rima pudendi. This manipulation I term the labial support.

In two instances my aged friend, Dr. T. F. Jones, has supported the labia majora with the view of protection to the perineum, while I made the delivery with forceps in primiparæ cases without any injuries to the perineum, although forceps was resorted to with reluctance for fear of perineal injuries. In other primaparæ cases where I have resorted to forceps without any assistance, in fifty per cent. the perineum was badly injured.

Very valuable time may be saved by placing the middle and index fingers in the anus, catching the brow, mouth or chin, and bringing the head forward just as the occipital protuberance is passing under the symphisis pubis without any hazard to the perineum, provided the labia majora be well supported. But this should be done at the declining of the pains, thereby taking advantage of muscular rigidity.

